# An innovative environmental tool to evaluate the sustainability of anthropogenic processes: the tetrahedron approach

**DOI:** 10.1007/s11356-024-34495-0

**Published:** 2024-08-01

**Authors:** Abdessamad Gueddari-Aourir, Carlos Alonso-Moreno, Jorge Enrique Zafrilla, Jesús Canales-Vázquez, María Concepción Ayuso-Yuste, Elena Villaseñor, Santiago García-Yuste

**Affiliations:** 1 Departamento de Química Inorgánica, Orgánica y Bioquímica-Centro de Innovación en Química Avanzada (ORFEO-CINQA), Universidad de Castilla-La Mancha, Avda. Camilo Jose Cela, 10, 13071 Ciudad Real, Spain; 2 Unidad nanoDrug, Facultad de Farmacia de Albacete, Universidad de Castilla-La Mancha, Av. Dr. Jose Maria Sánchez Ibáñez s/n, 02071 Albacete, Spain; 3 Departamento de Análisis Económico y Finanzas, Facultad de Ciencias Económicas y Empresariales, Universidad de Castilla-La Mancha, Plaza de La Universidad, 1, 02071 Albacete, Spain; 4 Instituto de Energías Renovables, Escuela Técnica Superior de Ingenieros Industriales, Universidad de Castilla-La Mancha, Paseo de La Investigación, 1, 02071 Albacete, Spain; 5 Ingeniería del Medio Agronómico y Forestal, Producción Vegetal, Escuela de Ingenierías Agrarias, Universidad de Extremadura, Avda. Adolfo Suárez S/N, 06007 Badajoz, Spain; 6 Instituto Universitario de Investigación de Recursos Agrarios. Universidad de Extremadura, Avda. de Elvas, S/N, 06071 Badajoz, Spain; 7 Departamento de Química Inorgánica, Orgánica y Bioquímica, Facultad de Ciencias y Tecnologías Químicas, Universidad de Castilla-La Mancha, Campus Universitario, 13071 Ciudad Real, Spain

**Keywords:** Sustainability, Anthropogenic processes, Waste prevention, Waste management, Quantitative metric, Green chemistry, Circular economy, Tetrahedron Parameter Global Evaluator

## Abstract

The Tetrahedron approach is a new environmental tool adapted to assess the sustainability of anthropogenic processes. This tool is based on a four-step methodology that includes (a) the identification of critical parameters, (b) evaluation through the Tetrahedron Parameter Global Evaluator, (c) construction of a tetrahedron diagram based on the final scores and (d) quantitative estimation of the global sustainability. The Tetrahedron incorporates various aspects of sustainability, including economic, social and environmental factors, and provides a comprehensive framework for evaluating the impact of human activities. This article presents the methodology and application of the Tetrahedron in determining the sustainability of five case studies: CO_2_ capture, unconventional methanol production, the Solvay process, CO_2_-alcoholic fermentation process strategy and the CO_2_-Rumen fermentation process strategy. The results demonstrate the Tetrahedron as an effective and reliable tool to quantify the sustainability of anthropogenic processes and to promote sustainable practices across various industries and sectors. The Tetrahedron offers several advantages over other environmental assessment tools, including holistic approach, simplicity and flexibility.

## Introduction

**Fig. 1:**
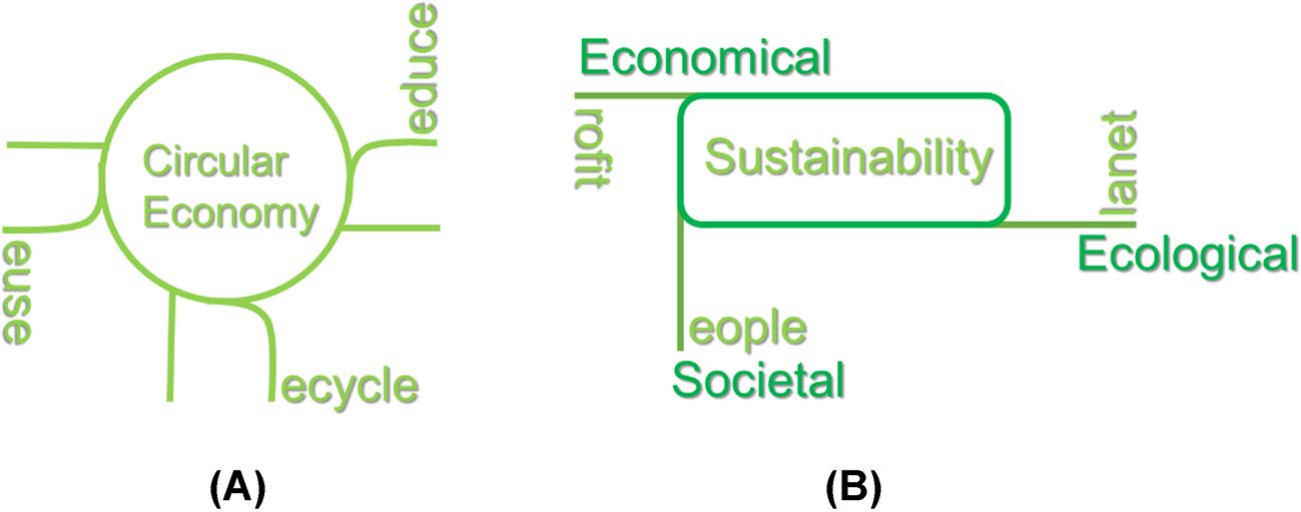
**A** Circular economy diagram based on 3R’s principle. **B** The sustainable diagram based on the 3P’s principle (IPCC 2014)

The concepts of sustainable development and circular economy (CE) have been on the agendas of a relevant number of countries on a global scale in recent years (UN 2015). Both concepts seem to be interrelated, as the circular model is believed to contribute to achieving various sustainable development goals (SDGs). Regarding the CE’s European Union model, the transition towards a more circular model intends the promotion of environmentally friendly actions, simultaneously ensuring sustainable economic growth and supporting job creation, aligning with a considerable number of SDGs (Schöggl et al. 2020). However, the literature indicates that the link between these concepts is somewhat ambiguous. Kirchherr et al. (2017) analysed 114 CE definitions to conclude that “the CE’s link to sustainable development is weak” (Kirchherr et al. 2017). To link both terms, Geissdoerfer et al. (2017) redefined them as follows: CE is a regenerative system in which resource input and waste, emission and energy leakage are minimised by slowing, closing and narrowing material and energy loops, and sustainability is the balanced integration of economic performance, social inclusiveness and environmental resilience, to the benefit of current and future generations. In this context of a certain conceptual lack of clarity, Ghisellini et al. (2016) presented the CE framework following the 3R’s principle: reduce, reuse and recycle (Fig. 1A). Recently, Panchal et al. (2021) proposed a bottom-up strategy to complete the diagram by including several key areas such as *production*, based on energy conservation and emission reduction technologies that are indirectly related to the best available technology (BAT); *consumption*, considering the choice of consumers, the environmental product footprint and the economic incentives of a product obtained under CE conditions; *waste management*, remarking the process of collection, transportation and disposal of waste; *secondary raw materials*, from waste management processes as an alternative to “virgin raw material”; *innovation and investments*, to enhance the research and development using new technologies and the concept of *sustainability*, based on the 3P’s pillars: people, profit and planet (Panchal et al. 2021). Thus, this diagram was designed to define an ideal sustainable process (Fig. 1B) (IPCC 2014).

Carbon-intensive industries must identify synergies between the CE model and the transition towards a more sustainable performance of the production and consumption patterns (Xie et al. 2023). This paper focuses on the chemical industry as one of the largest industrial energy consumers and the third-largest industry subsector in terms of direct CO_2_ emissions (Eurostat 2023; IEA 2024). Chemical processes are relevant anthropogenic activities that demand measurement models for sustainability assessment. In the early 90 s, the atomic economy, AE, and the E-factor were introduced as pertinent concepts for measuring sustainability (Sheldon 2007; Trost 1991). Some years later, Anastas published the *Twelve Principles of Green Chemistry* (TPGC) that provided a sustainable framework in chemistry through the use of raw materials in safe and efficient chemical transformation processes to minimise waste materials (Anastas 2007, Anastas and Warner 1998) and effectively link sustainability and chemistry. In 2017, Sheldon correlated chemistry, sustainability and CE by employing the E-factor (Sheldon 2017). The E-factor is calculated for a specific product, production site or even for an entire company, with the ideal E-factor value close to zero. Low E-factor values indicate lower manufacturing costs and toxic waste disposal, in addition to lower energy demand. Simultaneously, Anastas introduced the ‘Green ChemisTree’, a metaphorical design for modern environmental chemistry that identifies the TPGC as branches, depicting the BAT of the GC (i.e. mechanisms, procedures and design guidelines), and including green metrics such as AE and E-factor as leaves. Recently, Anastas and Zimmerman (2019) described *The Periodic Table of the Elements of Green and Sustainable Chemistry*, reviewing approaches to achieving green and sustainable chemistry through innovative chemistry, and the pursuit of sustainability goals (Sheldon 2017).

The concept of life cycle assessment, LCA, emerged to give significance to waste management which undoubtedly affects the environmental impact of any anthropogenic process across all stages of the product life (i.e. raw materials, production, distribution, disposal and/or recycling) (Sheldon 2017). In this context, Sheldon (2018) correlated sustainable development, SD, and CE recognizing the relevant role of various other terms in accurately assessing the sustainability of anthropogenic activities, such as the warming effect of greenhouse gases, GHGs, or the global climate change (Keijer et al. 2019, Sheldon and Brady 2022). Regarding the relation between the polluted Anthropogenic Age and CO_2_ atmospheric emissions, Poliakoff et al. (2015) coined the acronym CO_2_ CHEMISTRY to present the *Twelve Principles of CO*_*2*_* Chemistry in carbon dioxide utilisation (CDU) processes* (Poliakoff et al. 2015), which are based on the TPGC (Anastas 2007). In this regard, Sheldon proposed the concept of a SE, which considers waste management and CE components as the main parameters: raw material (Rm); production (P); consumption (C); waste prevention (Wp) and waste management (Wm) (Sheldon 2017, 2018). To expand the scope of sustainability in LCAs, a new concept that represents chemistry through circular process approaches has been recently introduced: circular chemistry (CC) that replaces the old-fashioned linear economy concept (LE) (Keijer et al. 2019). While CC has its specific principles, they are also based on the classical TPGC.

The main metrics reported to assess the sustainability of an anthropogenic process have been reviewed by Constable et al. (2002). The authors concluded that there is no unified metric available to measure the ‘greenness’ of anthropogenic processes yet and, therefore, fostered the need for strategic analysis through continuous improvements (Constable et al. 2002; Jiménez-González et al. 2012). In this sense, McElroy et al. (2015) reported a unified metric toolkit to evaluate the sustainability of anthropogenic processes by monitoring three new metrics: optimum efficiency (OE), renewable percentage (RP) and waste percentage (WP). Unfortunately, the sustainability of an anthropogenic process remains a challenge (García-Barragán et al. 2019; Jiménez-González et al. 2012; McElroy et al. 2015).

**Fig. 2:**
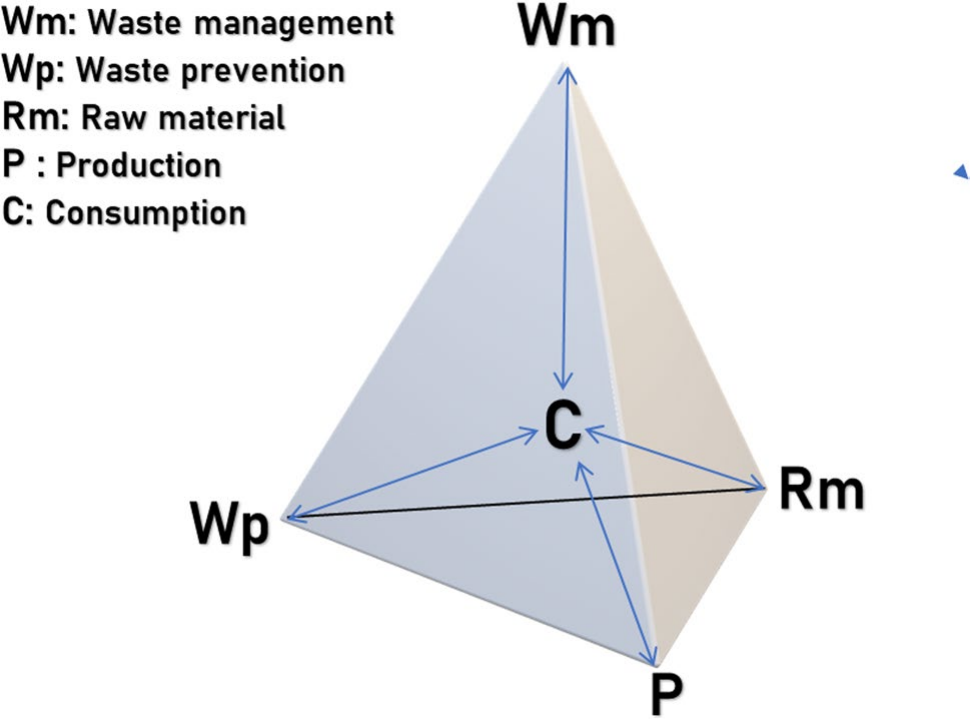
The *Tetrahedron* as a tool for assessing any anthropogenic process by employing circular economy parameters

This paper proposes the idea of the Tetrahedron (Td) as an environmental tool to evaluate the sustainability of any anthropogenic process (Fig. 2) and highlighting the relevance of the CE parameters mentioned above (Rm, P, C, Wp and Wm) (Alonso-Moreno & García-Yuste 2020). The current environmental situation demands all the CE parameters being considered simultaneously for an adequate assessment of the sustainability in any anthropogenic activity (Alonso-Moreno and García-Yuste 2020, Panchal et al. 2021; Sheldon 2018). The symmetry of the Tetrahedron, with four vortexes and a centroid element, correlates all CE parameters in a balanced relationship framework compared to the progressive CE diagram (Alonso-Moreno and García-Yuste 2020). A highly symmetric Td corresponds to a perfect equilibrium situation between all the CE parameters involved in an anthropogenic process. Herein, as a proof of concept, we assess the Td as a new holistic toolbox by quantifying the sustainability of some CDU processes.

## Materials and methods

### Definition of the Tetrahedron tool

**Fig. 3:**
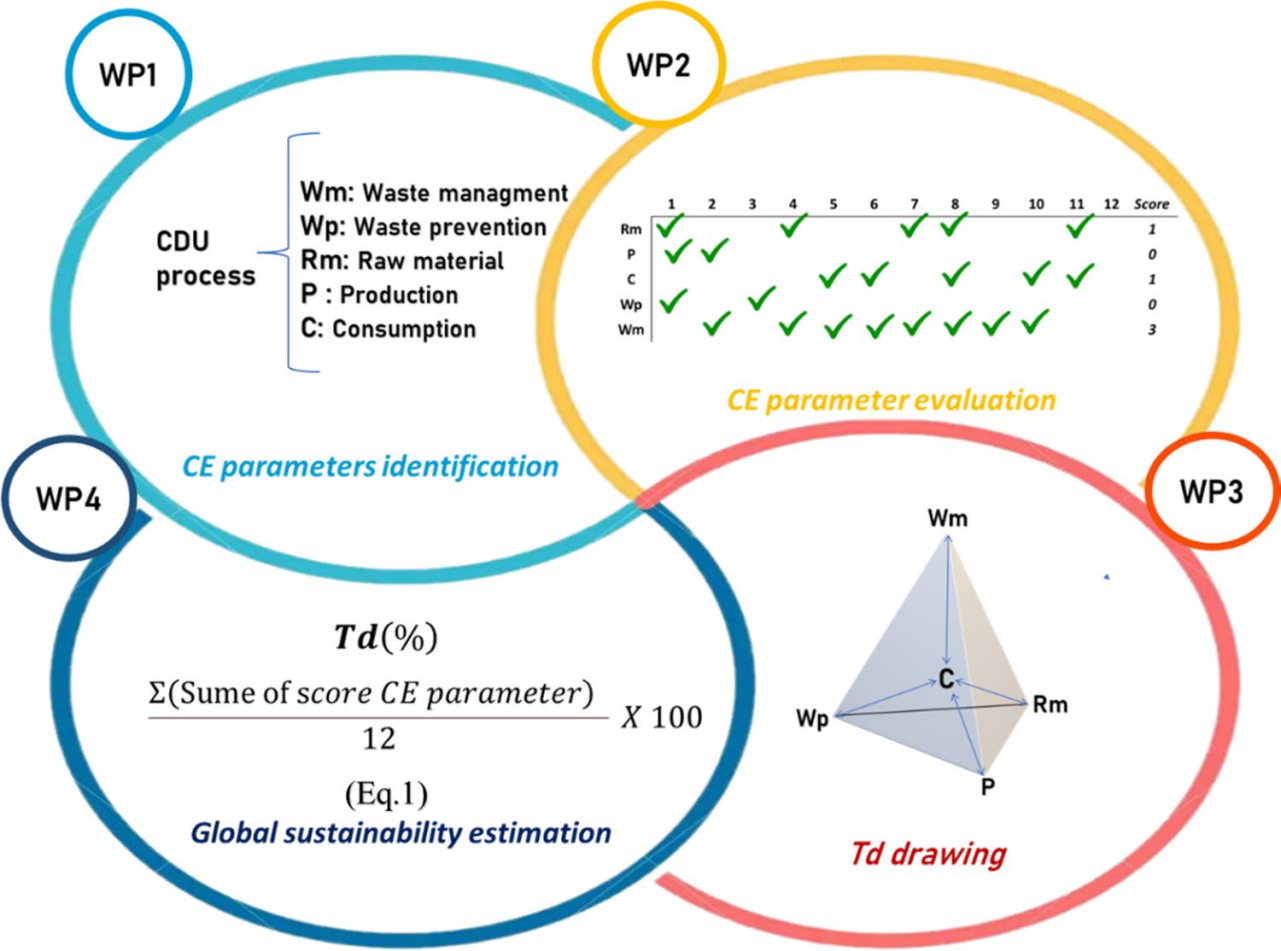
Methodology illustration for assessing the sustainability of anthropogenic activity using the Tetrahedron

The Td approach follows four work packages (WP) (Fig. 3):

1. WP1, a previously reported CDU process is analysed to identify the CE parameters (Table 1)
2. WP2, CE parameters based on anthropogenic activities are evaluated through the TPGC to give rise to a global score (Table 2). The global score is in the 0–3 range depending on the number of TPGCs fitting a CE parameter. 0 to 3 TPGCs correspond to a score of 0 (worst), 4–6 to a score of 1 (bad), 7–9 to a score of 3 (good) and finally 10–12 to a score of 4 (best) (Table 3)
3. WP3, a Td diagram is represented based on the final CE parameter scores. The symmetry of the Td enables the quantification of the sustainability of the anthropogenic processes under evaluation
4. WP4, the global sustainability is quantitatively estimated (Td (%),sustainability (%)) using Eq. 1 (see Eq. 1 in WP4 of Fig. 3)

**Table 1:** Circular economy parameters and description of the anthropogenic activity of the scientific article

CE parameter	Description of the anthropogenic activity in the scientific article
*Raw material*	Reagents and solvents employed
*Production*	Chemical synthetic methods (reaction conditions, products, methods, and technics described)
*Consumption*	The scientific article, (objectives, results, consequences)
*Waste prevention*	By-products generated and energy required
*Waste management*	Treatment to reduce the Wp effects

**Table 2:** The Twelve Principles of Green Chemistry, TPGC

1 Prevention: It is better to prevent waste than to treat or clean up waste after it has been created.
2 Atom economy: Synthetic methods should be designed to maximize the incorporation of all materials used in the process into the final product.
3 Less hazardous chemical syntheses: Wherever practicable, synthetic methods should be designed to use and generate substances that possess little or no toxicity to human health and the environment.
4 Designing safer chemicals: Chemical products should be designed to affect their desired function while minimizing their toxicity.
5 Safer solvents and auxiliaries: The use of auxiliary substances (e.g. solvents, separation agents and others) should be made unnecessary wherever possible and innocuous when used.
6 Design for energy efficiency: Energy requirements of chemical processes should be recognized for their environmental and economic impacts and should be minimized. If possible, synthetic methods should be conducted at ambient temperature and pressure.
7 Use of renewable feedstocks: A raw material or feedstock should be renewable rather than depleting whenever technically and economically practicable.
8 Reduce derivatives: Unnecessary derivatization (use of blocking groups, protection/deprotection, temporary modification of physical/chemical processes) should be minimized or avoided if possible because such steps require additional reagents and can generate waste.
9 Catalysis: Catalytic reagents (as selective as possible) are superior to stoichiometric reagents.
10 Design for degradation: Chemical products should be designed so that at the end of their function they break down into innocuous degradation products and do not persist in the environment.
11 Real-time analyses for pollution prevention: Analytical methodologies need to be further developed to allow for real-time, in-process monitoring and control prior to the formation of hazardous substances.
12 Inherently safer chemistry for accident prevention: Substances and the form of a substance used in a chemical process should be chosen to minimize the potential for chemical accidents, including releases, explosions and fires.

**Table 3:** Description of the anthropogenic activity together with the following parameters: raw material (Rm); production (P); consumption (C); waste prevention (Wp) and waste management (Wm)

Case	Rm	P	C	Wp	Wm^a^
1	K(OH)_(aq)_	DAC process	To capture CO_2_ for fuel production or sequestration process	No by-products	
2	CO_2(g)_, H_2(g)_, C catalyst	Direct reaction of pure reagents	To produce MetOH	O_2_ by-product	
3	NaCl-brine, CaCO_3_ NH_3(g)_	Produce Soda Ash	Traditional industrial method	–-	–-
4	CO_2(g)_, Na(OH) 50%wt	Na_2_CO_3_(aq) The *CO*_*2*_*-AFP strategy*	To capture CO_2_ and produce Soda Ash	Only H_2_O by-product	
5	CO_2_ enriched air and biogenic-NH_3(g)_	NH_4_HCO_3_(aq) by the *CO*_*2*_*-RFP strategy*	To produce NH_4_HCO_3_(aq) in a dairy farm	No by-products	

^a^Wm methods in cases 1, 2, 4 and 5 are not required due to the lack of waste products. Case 3 does not consider Wp and Wm procedures

### Scientific articles selection

Next, several scientific articles (indexed in the *Web of Science* database) associated with CDU processes have been selected as proof of concept of the Td methodology.

- Case 1: a process for capturing CO_2_ from the atmosphere in an industrial plant, using Direct Air Capture methodologies (DAC). The removed CO_2_ would be employed in fuel synthesis or sequestration processes (Keith et al. 2018).
- Case *2*: a non-conventional methanol production, direct conversion of CO_2_ to methanol and concomitant production of hydrogen from water electrolysis on a large scale in a CDU process (Marlin et al. 2018).
- Case 3: the traditional synthetic method to produce Soda ash (Na_2_CO_3_), i.e. the Solvay process, is discussed from an environmental point of view (Steinhauser 2008). This article was selected to properly compare results with those obtained from case 4.
- Case 4: a novel CDU approach based on the capture of CO_2_ from alcoholic fermentation processes (AFP). This strategy would employ extremely pure CO_2_ from the AFP process and NaOH aqueous solutions to produce one of the top 10 commodities in the chemical industry, Na_2_CO_3_ (Alonso-Moreno and Garcia-Yuste 2016).
- Case 5: an original CDU approach to reduce the NH_3_ emissions from a relatively minor biogenic carbon dioxide emission source, the ‘CO_2_-RFP strategy’, exploring the potential treatment of the liquid manure storage located at the bottom of the dairy barns with CO_2_-enriched air coming from cattle exhalation and belching to produce an important fertiliser such as NH_4_HCO_3_ (Alonso-Moreno et al. 2018).

### Circular economy parameters

Table 1 collects the CE parameters, and each study case is referred to a particular anthropogenic activity. Thus, reagents and solvents are identified as Rm, the chemical synthetic methods as production, P; objectives, methods, results and consequences of the scientific article as consumption, C; Wp, related to environmental concerns, especially by-products generated and energy employed, and the Wm, to reduce the Wp generated in the anthropogenic activity.

### Tetrahedron elaboration

The greener quality of a chemical process can be simply analysed by applying the Twelve Principles of Green Chemistry (TPGCs) and the circular economy (CE) parameters in the Td elaboration. Table 2 lists the TPGC (Anastas 2007, Anastas and Warner 1998). As mentioned above, every CE parameter is evaluated through the TPGC. When a CE parameter matches one of the TPGCs, a green tick (✓) is assigned; see Table 3. The final score has been labelled as *Worst*, 0; *Bad*, 1; *Good* 2 or *Best*, 3, respectively, as mentioned above.

To represent the Td (see Fig. 2), a CE parameter is placed at the centroid position, whereas the remaining CEs will be at the four vortexes. An ideal regular Td would be obtained when each of the vortexes had a value of 3 (see equation in WP4 of Fig. 3).

## Results and discussions

### Case 1: A direct air capture methodology in an industrial plant

**Scheme 1:**
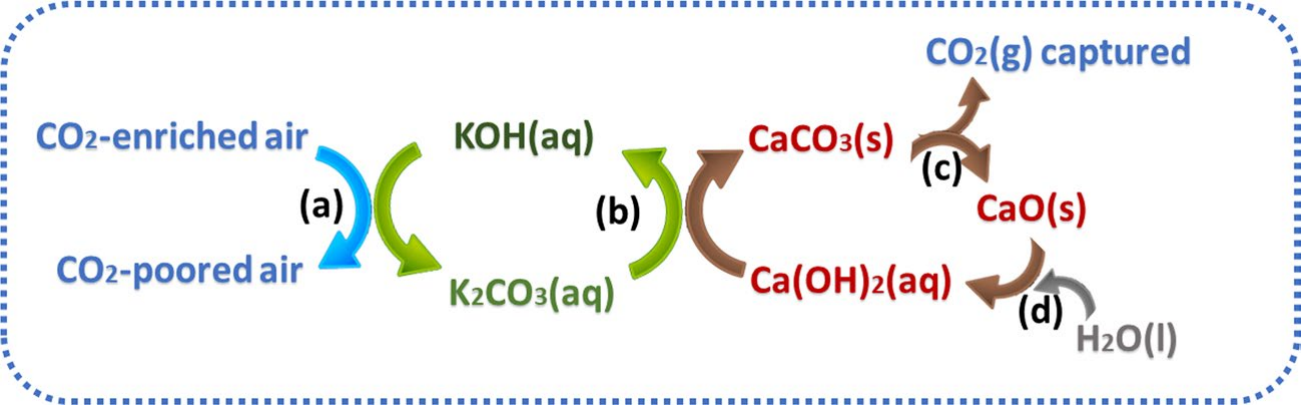
The direct air capture process: **a** CO_2_ removed from air and K_2_CO_3(aq)_ formation; **b** KOH regeneration and CaCO_3_(s) formation; **c** thermal decomposition of CaCO_3_(s); **d** Ca(OH)_2(aq)_ regeneration

The DAC process evaluated by Keith et al. (2018) refers to CO_2_ removal from the atmosphere in an industrial pilot plant and is considered as an air-clean process. The process captures continuously c.1 Mt-CO_2_/year using an aqueous KOH sorbent coupled to a calcium caustic recovery loop; see Scheme 1.

Table 3 compiles Rm, P, C, Wp and Wm parameters and the description of the anthropogenic activity. The atmospheric CO_2_ is captured from the air by the following steps: (a) the atmospheric CO_2_ is transformed into K_2_CO_3(aq)_, by the use of an aqueous KOH solution; (b) K_2_CO_3(aq)_ reacts with Ca(OH)_2_(s) to regenerate KOH_(aq)_ and precipitate CaCO_3(s)_; (c) thermal decomposition of CaCO_3(s)_ to yield CaO_(s)_ and CO_2(g)_ as final products; (d) CaO_(s)_ reacts with H_2_O_(l)_ to produce Ca(OH)_(s)_ and reuse in step (b). The CO_2(g)_ captured in step (c) would be used in other CDU or CO_2_ sequestration processes. All the steps correspond to very high–yield reactions. Additionally, the energy demands in steps (a–d) of the DAC pilot plant were optimised, and small amounts of hazardous by-products were obtained in the global process. Intentionally, KOH_(aq)_ corresponds to a very well-known compound in industry and the only solvent was H_2_O_(l)_. Finally, the DAC methodology is monitored in real time to control the global process and minimise accident risk and pollution. All the above suggests that the CE parameter (Rm, P, C and Wp) matches most of the TPGC (see analysis in Table 4), whilst no Wm is identified. This CDU process would be considered as an integral waste management process of CO_2_-enriched atmospheric air.

**Table 4:** Estimation of circular economy parameters through TPGC for cases 1–5

CE parameter	Twelve Principles of Green Chemistry												Score^a^
Case 1: A DAC methodology in an industrial plant													
	**1**	**2**	**3**	**4**	**5**	**6**	**7**	**8**	**9**	**10**	**11**	**12**	
Rm	✓		✓		✓	✓		✓			✓	✓	**Good, 2**
P	✓		✓	✓	✓	✓		✓			✓	✓	**Good, 2**
C	✓		✓	✓	✓	✓		✓			✓	✓	**Good, 2**
Wp	✓		✓	✓	✓	✓		✓			✓	✓	**Good, 2**
Wm	✓		✓	✓	✓	✓		✓			✓	✓	**Good, 2**
Case 2: MeOH synthesis by a catalytic method													
	**1**	**2**	**3**	**4**	**5**	**6**	**7**	**8**	**9**	**10**	**11**	**12**	
Rm	✓		✓	✓	✓	✓		✓	✓		✓	✓	**Good, 2**
P	✓		✓	✓	✓	✓		✓	✓		✓	✓	**Good, 2**
C	✓		✓	✓	✓	✓		✓	✓		✓	✓	**Good, 2**
Wp	✓		✓	✓	✓	✓		✓	✓		✓	✓	**Good, 2**
Wm	✓		✓	✓	✓	✓		✓	✓		✓	✓	**Good, 2**
Case 3: The Solvay process													
	**1**	**2**	**3**	**4**	**5**	**6**	**7**	**8**	**9**	**10**	**11**	**12**	
Rm											✓	✓	**Worst, 0**
P											✓	✓	**Worst, 0**
C											✓	✓	**Worst, 0**
Wp													**–-**
Wm													**–-**
Case 4: The CO_2_-AFP strategy													
	**1**	**2**	**3**	**4**	**5**	**6**	**7**	**8**	**9**	**10**	**11**	**12**	
Rm	✓	✓			✓	✓	✓	✓				✓	**Good, 2**
P	✓	✓	✓	✓	✓	✓		✓				✓	**Good, 2**
C	✓	✓	✓	✓	✓	✓	✓	✓				✓	**Good, 2**
Wp	✓	✓	✓	✓	✓	✓		✓				✓	**Good, 2**
Wm	✓	✓	✓	✓	✓	✓		✓				✓	**Good, 2**
Case 5: The CO_2_-RFP strategy													
	**1**	**2**	**3**	**4**	**5**	**6**	**7**	**8**	**9**	**10**	**11**	**12**	
Rm	✓		✓	✓	✓	✓	✓	✓		✓		✓	**Good, 2**
P	✓		✓	✓	✓	✓	✓	✓		✓		✓	**Good, 2**
C	✓		✓	✓	✓	✓	✓	✓		✓		✓	**Good, 2**
Wp	✓		✓	✓	✓	✓	✓	✓		✓		✓	**Good, 2**
Wm	✓		✓	✓	✓	✓	✓	✓		✓		✓	**Good, 2**

^a^For each CE parameter, the total quantification is scaled from 0 to 3. Accordingly, when the CE parameter fits in with 0 to 3; 4 to 6; 7 to 9 or 10 to 12 of the TPGC, the score would be *worst*, 0; *bad*, 1; *good* 2 and *best*, 3, respectively

**Fig. 4:**
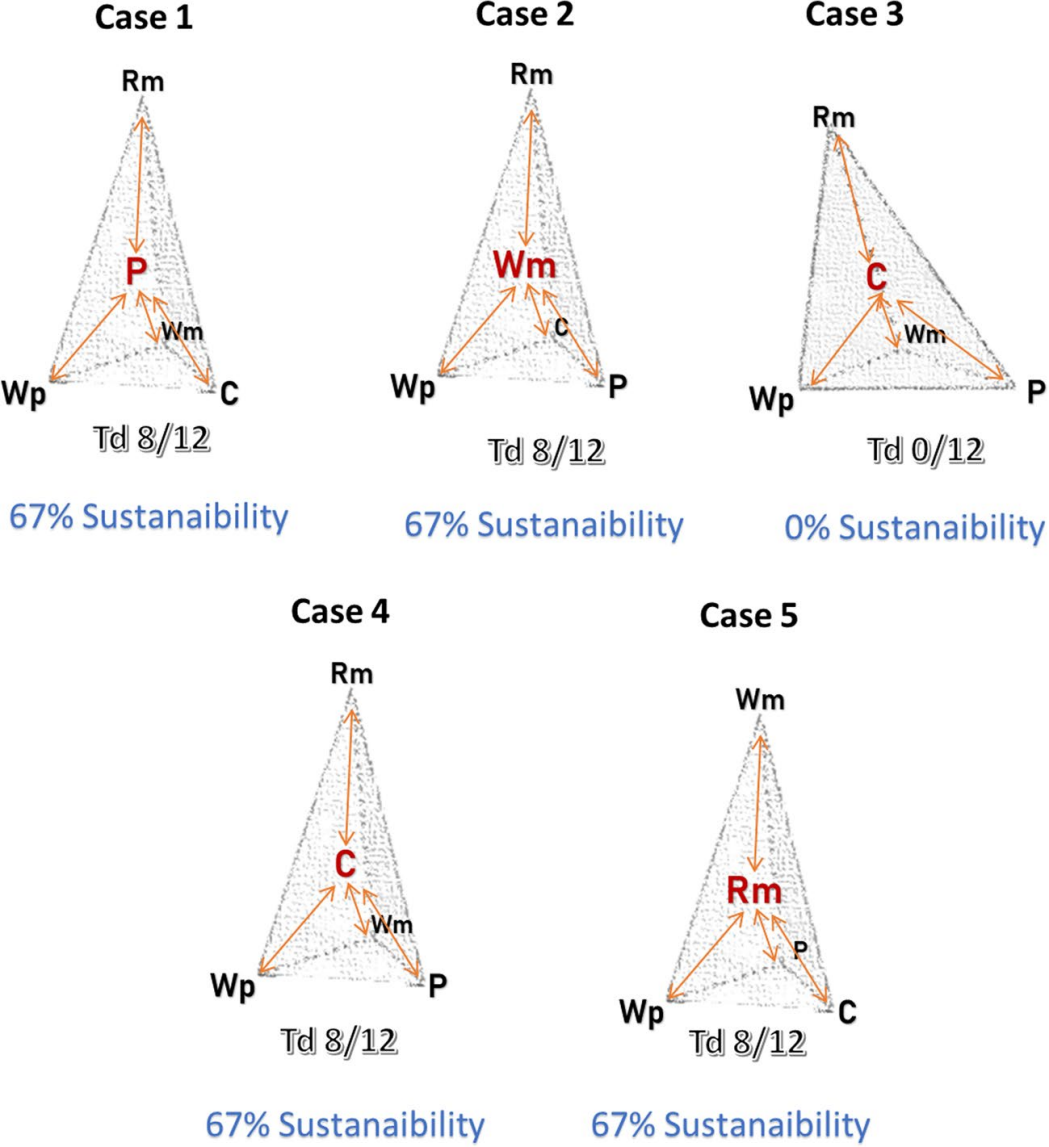
Tetrahedron shape and % sustainability for cases 1–5

Based on the Td metric calculated for this process using all the CE parameters (see Table 4), an 8/12 Td is depicted, as shown in Fig. 4. After applying Eq. 1, the %Td sustainability is determined to be 67%.

### Case 2: MeOH synthesis by a catalytic process in the Carbon Recycling International geothermal power plant in Iceland

**Scheme 2:**
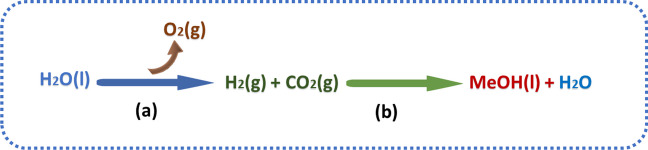
Methanol synthesis in the Recycling International geothermal power plant in Iceland. **a** Water; (**b**) catalytic process

Case 2 corresponds to a sustainable methanol synthesis by a catalytic process carried out in the Carbon Recycling International geothermal power plant in Iceland (see Table 3 for the CE parameters and Scheme 2 for the illustration of the activity) (Marlin et al. 2018). The use of pure CO_2(g)_ and H_2(g)_ and a catalyst (the most adequate catalyst for this kind of processes: copper oxide, zinc oxide and alumina (CZA)) implies a very low energy profile for the process. The only by-products are O_2_, H_2_O and unreacted CO_2_.

This method for methanol synthesis simplifies the chemistry requirements and improves the purification processes in conventional methanol industrial plants (Marlin et al. 2018). The main advantage is that the reaction impurities are essentially water and unreacted CO_2_, both dissolved in the crude methanol product, giving both economic and environmental benefits versus the *syn-gas* process (Marlin et al. 2018). Moreover, Case 2 uses renewable sources for electricity generation under mild conditions to prevent waste, and the use of a catalyst ensures faster syntheses. The process is continuously monitored to decrease the CO_2_ concentration in the atmosphere. Since most of the CE parameters align with the TPGC (as detailed in the analysis in Table 4), Fig. 4 illustrates an irregular Tetrahedron with a %Td sustainability value of 67%.

### Case 3: The Solvay process

**Scheme 3:**
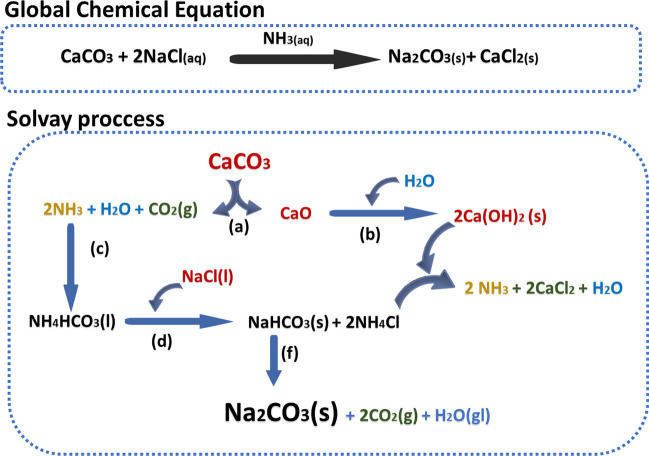
Solvay process adapted diagram (Steinhauser 2008): **a** thermal decomposition of CaCO_3_; **b** Ca(OH)_2_ formation; **c** NH_3_ absorption; **d** NaCl-brine reaction with NH_4_HCO_3_; **e** NH_3_ recovery; **f** soda ash formation

The elementary global chemical equation of the Solvay process is depicted in Scheme 3 (Steinhauser 2008). It is essential to note that this process is highly energy-demanding, involving the manipulation of chemicals such as NH_3_ or CO_2_. Furthermore, the decomposition of CaCO_3_(s) is also a high-energy step (see CE parameters for case 3 in Table 3). After analysing the circular economy parameters in the search for matches with the TPGC, it is evident that the Solvay process cannot be classified as sustainable. This alignment occurs only for principles 11 and 12, which fit with the Rm, P and C parameters (see Table 4 for CE parameter evaluation).

Therefore, this process shows an asymmetric 0/12 Td with a %Td sustainability value of 0, fitting in with a traditional LE framework (raw material → production → consumption) (Alonso-Moreno and García-Yuste 2020), which corresponds to a non-sustainable process.

### Case 4: The CO_2_-AFP strategy

**Scheme 4:**
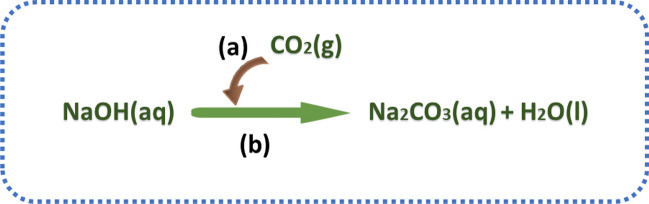
The CO_2_-AFP strategy chemical process. **a** CO_2_ emitted by AFP. **b** The reaction is taking place in a carbonator tank

This strategy reported the CO_2_ capture from alcoholic fermentation processes (Alonso-Moreno and Garcia-Yuste 2016). Later, the strategy was successfully scaled up (López Montero et al. 2022), and the carbon footprint balance of a real-case wine fermentation CO_2_ capture was calculated (Gueddari-Aourir et al. 2022). The CO_2_-AFP strategy is a CDU approach from a relatively minor CO_2_ emission source, such as alcoholic fermentation processes (AFPs). The extremely pure CO_2_ from the AFP is captured in NaOH solutions to produce Na_2_CO_3_. This process consumes less energy than the Solvay soda process and does not produce low-value by-products (see Scheme 3 for the global reaction and Table 3 for the definition of the CE parameters) (Scheme 4).

The Rm, P, C and Wp parameters for the CO_2_-AFP strategy facilitate the prevention of by-products by a high AE process, where reagents and solvents are environmentally friendly and compatible with food industry. The design also displays energy efficiency (low temperature and atmospheric pressure), and no Wm is required. Consequently, most CE parameters align with the TPGC (see Table 4). However, the CO_2_-AFP strategy does not fit in with the 9, 10 and 11 principles in any of the CE parameters evaluated because the process does not use catalysts and the final product, Na_2_CO_3_, is not biodegradable. Then, the 8/12 Td has a value of 67% for sustainability, contrasting with the Solvay process in case 3. One notable difference between both cases is the absence of Wp and Wm parameters in case 3. Case 4 aligns with the ‘the end justifies the means’ principle from the current greener philosophy of TPGC (Alonso-Moreno and García-Yuste 2020, Anastas 2007; Phan et al. 2015).

Two different Td diagrams can be drawn for Na_2_CO_3_ production. On one hand, the CO_2_-AFP strategy exhibits a high degree of sustainability (Alonso-Moreno and Garcia-Yuste 2016). On the other hand, the traditional soda Solvay process (Steinhauser 2008; Thieme 2000) can be considered analogous to the LE diagram. This can be understood because the Solvay process (Thieme 2000) has been in operation for more than a century, before the TPGC definition (Anastas 2007).

### Case 5: The CO_2_-RFP strategy

**Scheme 5:**
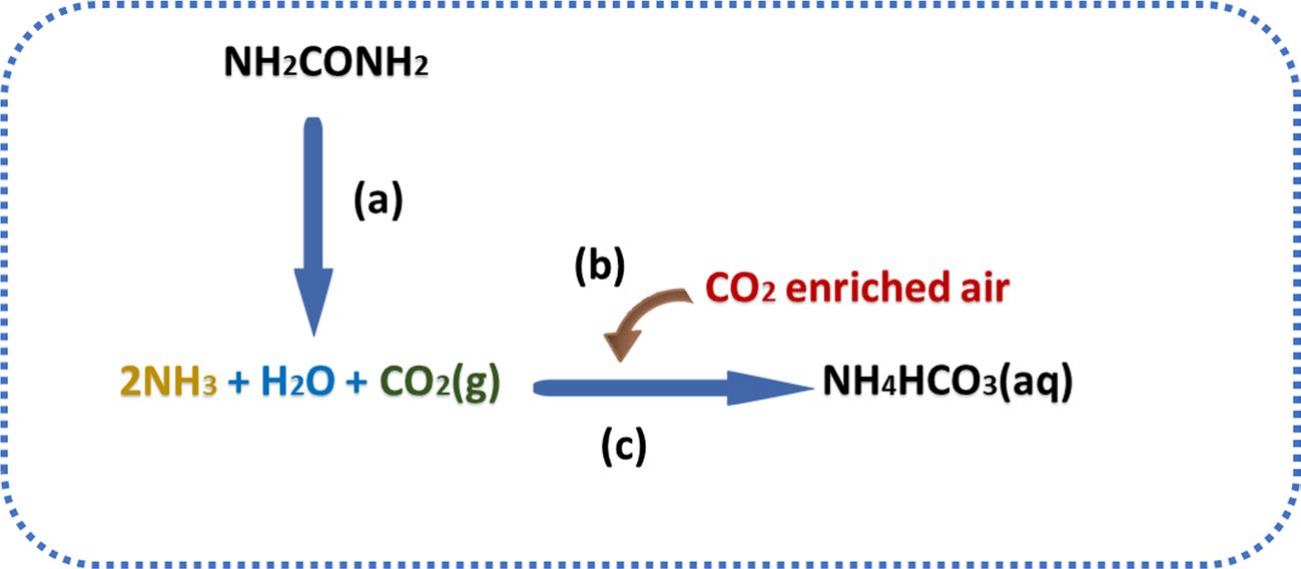
Global chemical process of the *CO*_*2*_*-RFP strategy*. **a** Urea hydrolysis; **b** CO_2_-enriched air coming from the cattle exhalation; **c** room temperature and atmospheric pressure

The CO_2_-RFP strategy (Alonso-Moreno et al. 2018), Rumen fermentation processes (RFP), explores the potential treatment of the liquid manure storage located at the bottom of the dairy barns with CO_2_-enriched air coming from cattle exhalation and belching to produce an important fertiliser such as NH_4_HCO_3_ (ABC, ammonium bicarbonate), in an exothermic process; see Scheme 5 for illustration and Table 3 for the description of CE parameters. The ABC fertiliser, a biodegradable product, is an environmental alternative to traditional N-fertiliser (urea, ammonium nitrate).

All reagents and solvents proposed in this anthropogenic activity aim to reduce NH_3_ emissions using the CO_2_ generated in the dairy farm. No extra water is necessary, and only biogenic reagents from a dairy farm are used. The amount of energy needed is low, as it operates under conditions of standard room temperature, atmospheric pressure and with minimal electricity expenses. Dairy production could ensure the existence of renewable raw materials for this process. This is a potentially safe chemical process to be implemented in a dairy farm because no extra reagents are needed, and recycling *biogenic* chemical compounds is produced on the farm. Then, the process prevents waste production because NH_4_HCO_3_ can be used as a *biogenic* fertilizer. Therefore, no Wm actions are required. Table 4 compiles the analysis of the CE parameters, resulting in an asymmetric Tetrahedron with a sustainability value score of 67%.

### Twelve principles of green chemistry and Tetrahedron main results

According to the analysis in Table 4, all the CDU processes implemented or under study show that the CE parameters align with most of the TPGC. This situation agrees with a high degree of sustainability in all cases as expected from their design. A few differences arose between *cases*; specifically, in the *CO*_*2*_*-RFP strategy* (Alonso-Moreno et al. 2018), only the 7-principle aligns because the *Rm* employed is constantly regenerated from the dairy metabolism. Another important difference between them is that the 10-principle fit occurs only in *case 5* due to NH_4_HCO_3_ being a biodegradable product. Additionally, *case 2* is the only catalytic process and therefore the one matching with the 9-principle.

### Strengths, weaknesses, opportunities and threats of Tetrahedron metric

The Td metric proves to be a useful and reliable qualitative and quantitative analysis tool. The CDU processes showed an anthropogenic activity close to the Brundtland and Sheldon sustainability definition (Sheldon 2018), ensuring the fulfilment of present-day needs without compromising the ability of future generations. All the CDU innovative environmental proposals protect the environment by simultaneously aligning with at least eight of the TPGC, all CE parameters. Analogously, this sustainability data grants the natural resources and residues consumed and generated at acceptable rates to the natural environment (Constable et al. 2002; Sheldon 2017). Moreover, we consider that the Td metric might be calculated for a particular product or production site from a chemical point of view. While achieving an ideal value of 100% sustainability may be utopic, we believe that a Td metric indicating sustainability close to 66% would indicate low manufacturing costs, responsible waste disposal and reduced energy demand, analogously to the E-factor (Sheldon 2017).

iSustain was reported in 2013, but it has not proven useful for the academic and industrial community in defining the sustainability concept, as mentioned in the “Introduction” section of this article (Koster and Cohen 2013). Therefore, to the best of our knowledge, this is the first environmental metric capable of quantifying the implementation of the TPGC. Furthermore, the Td metric is a candidate to be included as a new metrics box in the ‘The Periodic Table of the Elements of Green and Sustainable Chemistry’ (Anastas and Zimmerman 2019). Therefore, Td is a new sustainability concept, and a Td 7/12 or higher for a process implies that (i) at least seven of the TPGC align with each the CE parameter; (ii) natural resources are mainly preserved; (iii) residues are essentially minimised and (iv) the 3Ps Principle are considered in the design of the process (Ghisellini et al. 2016; Panchal et al. 2021).

Although having a good Td metric means being consistent with many of the 17 Sustainable Development Goals (SDGs) (Del Río et al. 2021) (i.e. clean water and sanitation, affordable and clean energy, responsible consumption and production, climate action, life below water, life on land) and ensures improvement of the remaining (SDGs) (Del Río et al. 2021), some critical points of this novel approach might be identified. Thus, strengths, weaknesses, opportunities and threats are compiled in Table 5, where strengths and opportunities are more significant than weaknesses and threats supporting the tool’s viability.

**Table 5:** SWOT analysis of Td metric

**Strengths**	**Weaknesses**
Quantitative metric	
Clarify the Sustainability concept	Dependence on chemistry knowledge
Waste prevention (substances, energy)	Influence by the CE parameters
Safeguard natural resources	Controlled by the TPGC
Harmony with TPGC	The minor concern of people directly
Friendly with AE and E-factor	The minor concern of profit directly
Agree with 3P’s Pillars Principle	
Friendly with SDGs	
**Opportunities**	**Threats**
Environmental metrics tool	
Descriptor of Sustainability Enhance the CE parameters	Focus on chemical analysis
Potential index of products and companies	Change CE vs sustainability
Enhance the F-factor of products	

## Conclusion

The alignment of chemical processes with the fundamental principles of green chemistry operating within a CE framework and parameters has shown remarkable results. A well-designed combination of these factors can lead to improved efficiency and reduced environmental impact compared to more conventional processes. The encouraging outcomes underscore the considerable potential of sustainable practices and their key role in driving a paradigm shift in the chemical industry. Benefits such as waste minimization and resource conservation can mitigate related environmental impacts and preserve ecosystems. Beyond immediate ecological concerns, the integration of green chemistry principles and CE can contribute to long-term sustainable growth as industries foster and internalize a culture of innovation.

Proper assessment and measurement of measures taken to improve environmental efficiency are keys for companies and institutions to drive significant sustainability changes. A thorough evaluation of the outcomes provides solid foundations for informed decision-making, allowing a better understanding of structural decisions. Embracing a measurable and transparent approach to environmental efficiency strengthens their social responsibility and strategically positions businesses to attract environmentally conscious consumers and business partners. In this sense, the proposed Td framework demonstrates remarkable efficacy as a tool for evaluating the sustainability of chemical processes, particularly in the context of carbon direct conversion (CDC). The findings and results presented in the paper strongly indicate that the widespread of the Td framework holds tremendous potential for enhancing sustainability assessments within the chemical industry. The interpretation of the Td results regarding environmental, social and economic impacts would aid in making better decisions leading to greener and more responsible practices.

Sustainable practices adherent to the principles of the CE present an opportunity to optimize the resource utilization in chemical processes significantly. Tools such as the Td would help industry professionals and policymakers to make informed decisions concerning the design and implementation of greener chemical processes. By heeding these insights, the industry would foster a future where the chemical industry thrived in harmony with human well-being and the environment.

## Data Availability

The data that support the findings of this study are available from the corresponding author upon reasonable request.
